# Symmetry-based analysis after surgical treatment of zygomaticomaxillary complex fractures using intraoperative cone-beam computed tomography: a retrospective case-control study

**DOI:** 10.1038/s41598-025-90481-7

**Published:** 2025-02-18

**Authors:** Adrian Franke, Jan Bernard Matschke, Michaela Bučkova, Lea Rahrisch, Günter Lauer, Henry Leonhardt

**Affiliations:** 1 Department of Oral and Maxillofacial Surgery, University Hospital Carl Gustav Carus Dresden, Technical University Dresden, Fetscherstraße 74, 01307 Dresden, Germany; 2 Intern Oral and Maxillofacial Surgeon, Department of Oral and Maxillofacial Surgery, University Hospital Carl Gustav Carus Dresden, Technical University Dresden, Fetscherstraße 74, 01307 Dresden, Germany; 3 Department of Oral and Maxillofacial Surgery, University Hospital Carl Gustav Carus Dresden, Technical University Dresden, Fetscherstraße 74, 01307 Dresden, Germany

**Keywords:** Zygomatic fracture, Maxillary fracture, Three-dimensional imaging, Cone-beam computed tomography (CBCT), Open fracture reduction, Internal fracture fixation, Outcomes research, Skeleton, Trauma

## Abstract

Zygomaticomaxillary fractures are among the most common fractures of the facial skeleton. Open reduction and internal fixation require radiographic control. Three-dimensional radiographs provide superior information on actual distances. The study aims to quantify and evaluate intraoperative reduction control by cone-beam computed tomography. The retrospective case-control study evaluates three-dimensional radiographs through linear measurements of defined skeletal landmarks from the median sagittal plane for symmetry. The study group received open reduction and internal fixation for zygomaticomaxillary fractures, and the control group consisted of a population without pathology of the midfacial region. The study group showed the same degree of symmetry as the control group. The mean absolute distance of all landmarks was 1.5 ± 1.3 mm in the study group and 1.0 ± 0.9 mm in the control group. There was a statistically significant likelihood of the right side being further away from the midline than the left. The study showed adequate reduction results of zygomaticomaxillary fractures. Moreover, the same degree of symmetry was ascertained compared to a control group. Intraoperative cone-beam computed tomography serves as a valid tool to check for immediate reduction control during surgery for zygomaticomaxillary fractures.

## Introduction

A significant proportion of trauma patients are affected by cranio-maxillofacial injuries that can present as isolated lesions or in combination with other serious injuries, such as spinal or upper and lower extremity injuries^1,2^. The population studied highly affects the epidemiology of facial fractures through a variation in type, severity, and cause^3,4^.

The zygoma and its anatomical subregions, collectively known as the zygomaticomaxillary complex (ZMC), form the lateral midface and are positioned on each side of the maxillary portions of the central midface pyramid. They transition into the greater wing of the sphenoid, frontal, and temporal bone^5^. ZMC fractures constitute between 17% and 60% of all skull fractures, depending on the geographic location where the study was conducted^6–8^. Socioeconomic, cultural, and environmental factors might be the leading causes of various trauma patterns^9^.

Open reduction and internal fixation (ORIF) are commonly performed to treat ZMC fractures. The main treatment principle is restoration of anatomical integrity and, ideally, symmetry of the midfacial region. Baseline fracture reduction control employs conventional X-ray imaging in two planes depicted by paranasal sinus view and “bucket handle” projections^10^. However, conventional 2D imaging methods often fall short due to the superimposition of bony structures, making precise evaluations challenging^11^, and are usually performed after the operation with the patient awake.

For intraoperative use, mobile X-ray devices, such as the C-arm, play a pivotal role in traumatology and orthopaedics surgery^12,13^. In maxillofacial surgery, intraoperative imaging developed from C-arms that can perform three-dimensional imaging^14–16^ over intraoperative computed tomography (CT)^17,18^ to modern cone-beam computed tomography (CBCT)^19–21^. Three-dimensional imaging techniques offer a significant advantage by allowing the detailed assessment of complex anatomical structures such as the orbit, mandibular condyle, and ZMC.

There is a plethora of studies that are concerned with the symmetry of the facial skeleton. Even though there is an apparent trend towards three-dimensional evaluation of the structure of the facial skeleton^22^, most institutions cannot perform complex three-dimensional analysis. A recently published work provides an easy-to-employ method to ascertain symmetry through linear measurements in CT scans with virtually any DICOM viewer^23^.

This work serves as a follow-up study for symmetry measurements of the facial skeleton. It aims to evaluate reduction controls after ORIF of ZMC fractures by intraoperative three-dimensional imaging. In particular, symmetry measurements are to be quantified, and a cohort of patients receiving ORIF of ZMC fractures will be compared to a control group of healthy patients.

## Methods

### Study design, setting, and participants

Following the STROBE (strengthening the reporting of observational studies in epidemiology) guidelines, the study was designed and implemented as a retrospective case-control study that examined intraoperative CBCT scans for symmetry after ORIF of ZMC fractures in an inpatient setting. Patients were treated by the Department of Oral and Maxillofacial Surgery at the University Hospital Carl Gustav Carus in Dresden, Germany, between November 2018 and December 2022. At random, any surgeon of the entire team performed surgeries. Registrars were supervised by an experienced consultant in oral and maxillofacial surgery. The AO Foundation’s level 2 midface classification system addresses the zygoma and the zygomatic arch as a single anatomic region, defined as the lateral midface^24^, or the ZMC. Inclusion criteria were (1) unilateral midface fractures according to AO Foundation’s Level 2 and (2) adequate imaging quality. Exclusion criteria were (1) bilateral midface fractures since the healthy sides provided information about the original state and were used to assess symmetry, (2) extensive or combined fractures involving the central midface or craniofacial fractures that would adversely affect the skeletal landmarks needed for measuring symmetry, and (3) inadequate imaging quality caused by artefacts due to the patient’s movement, blurred imaging for any other reason, incomplete imaging of the region of interest due to false positioning, or metallic foreign bodies, i.e., metallic dental materials or implants.

The control group was chosen randomly from a pool of individuals receiving a medically indicated CT scan of the midface in an inpatient or outpatient setting at the University Hospital Carl Gustav Carus in Dresden, Germany, between February 2016 and February 2022. Patients were included when there was (1) no bony destruction or any significant bone pathology of the skull and the midface, (2) sufficient imaging quality, and (3) full legal age. Scans were excluded when any asymmetry-producing pathologies like clefts, fibrous dysplasia, chronic sinusitis, midfacial injuries, or similar were evident. Participants in the control group were chosen to match the age distribution of the study group, ensuring that age-related factors influencing the outcome were evenly balanced. The ratio of males to females in the control group was chosen to be 1:1 to reflect the general population accurately. Additional factors, such as socioeconomic status, geographic location, and ethnicity, were not included in the matching process because the patient records did not provide enough information due to the study’s retrospective nature.

The study was conducted according to the guidelines of the Declaration of Helsinki. The Ethics and Institutional Review Board of the Technische Universität Dresden (institutional review board number IRB00001473) registered at the Office for Human Research Protections (IORG0001076) approved the study (internal ethics committee ID number: BO-EK-371082023). Due to the retrospective nature of the study, informed consent was waived.

### Variables, data measurements, and bias

**Fig. 1:**
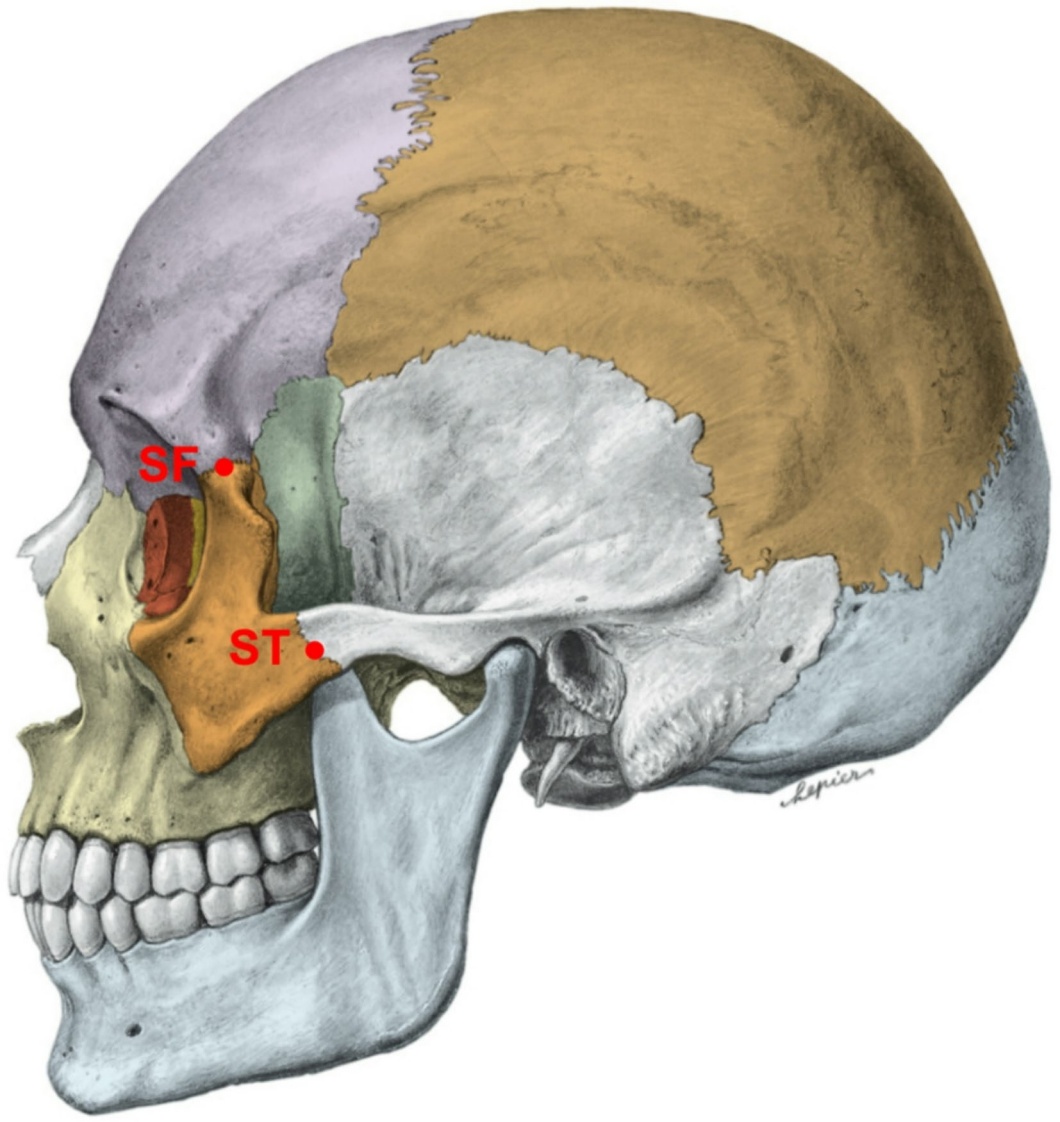
Lateral view of the skull. SF = frontozygomatic suture; ST = temporozygomatic suture.

**Fig. 2:**
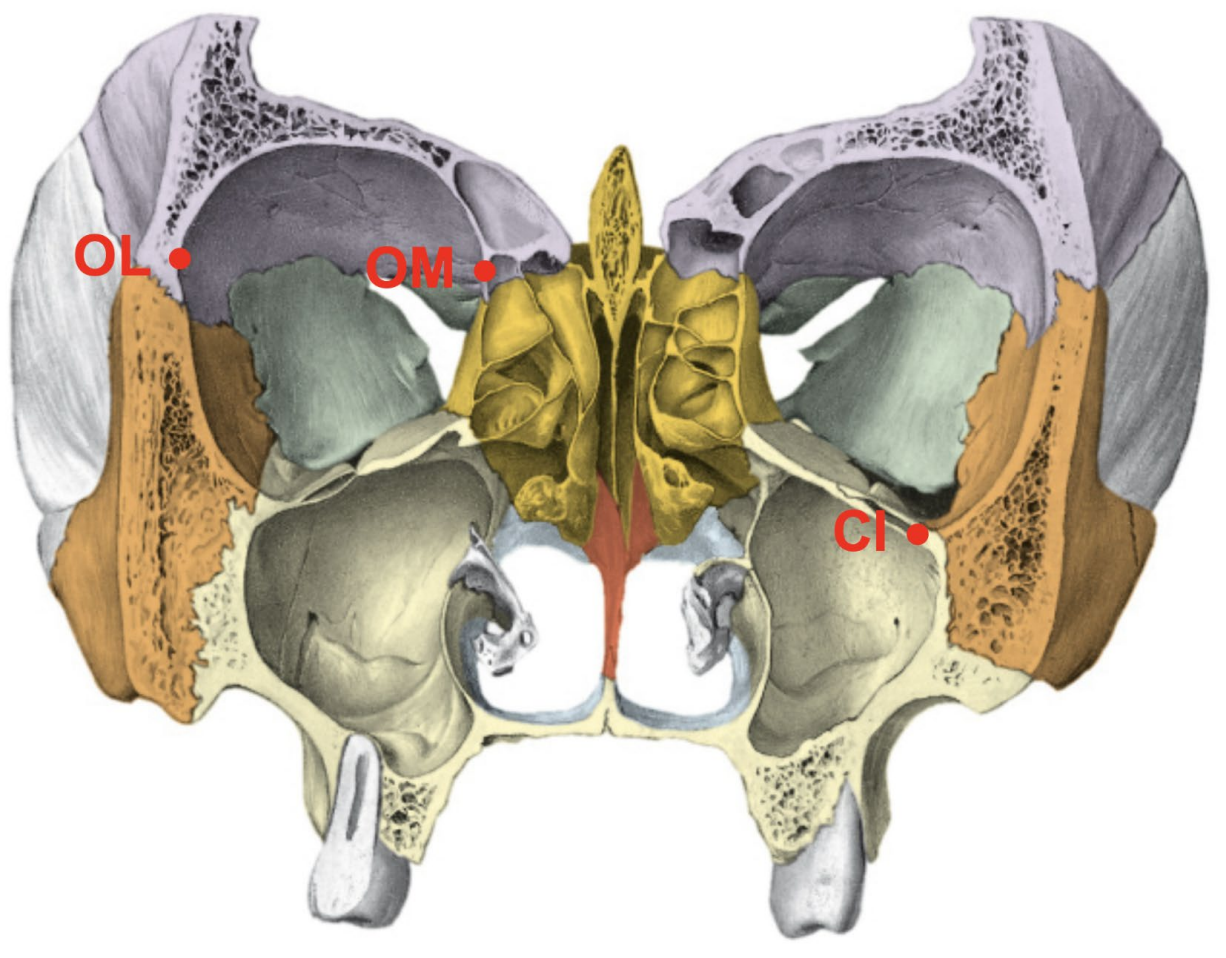
Coronary section of the skull. OL = lateral orbita, OM = medial orbita, CI = infraorbital canal.

**Fig. 3:**
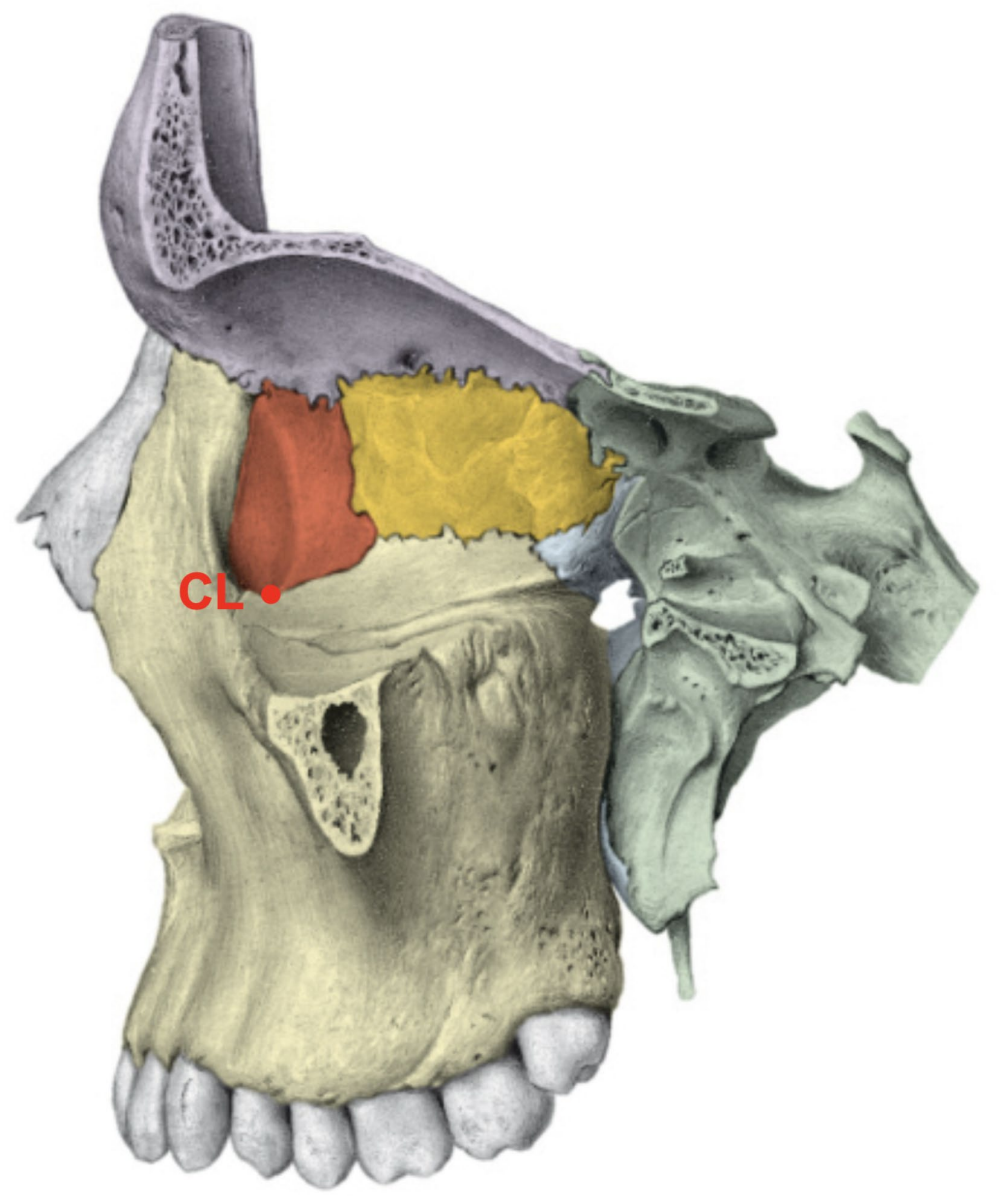
Sagittal section of the skull viewing the medial orbita. CL = crista lacrimalis posterior.

All patients’ sex and age at the imaging time were recorded. Six anatomical landmarks (frontozygomatic suture (SF), temporozygomatic suture (ST), infraorbital canal (CI), crista lacrimalis posterior (CL), lateral orbita (OL), and the medial orbita (OM)) were defined, and their distances were measured in millimetres (mm) from the median sagittal plane as described in the literature (Figs. 1, 2 and 3)^23^. The same study has already addressed bias, rendering another check for reproducibility obsolete.

The primary outcome was to find whether patients in a study group showed a symmetrical midface after ORIF of ZMC fractures. The secondary outcome was to compare the results to a healthy control group, verifying adequate ORIF.

The study group received an intraoperative CBCT scan using the XORAN xCAT device (Xoran Technologies, LLC., Ann Arbor, Michigan, USA). After the patient’s region of interest was placed within the scanner’s acquisition field, the “sinus scan” protocol was used to obtain the images. The parameters were set to 120 kV and 57.6 mAs. The DICOM datasets were subsequently transferred to the IMPAX server.

The database of patients treated in the Department of Oral and Maxillofacial Surgery at the University Hospital Carl Gustav Carus in Dresden, Germany, was screened for patients who received a CT scan for any indication that fit the inclusion criteria. The SOMATOM Definition Edge Scanner (Siemens Healthineers, Erlangen, Germany) obtained the control group’s data: viscerocranial spiral CT; 2 × 64 × 0.6 mm collimation and stored on the IMPAX server. Only slices of 1 mm and less were selected.

For data analysis, the IMPAX EE software (v20190821_0813, Agfa HealthCare N.V.) was utilized for DICOM image presentation, multiplanar reconstruction, and postprocessing. The “bone windows” were used in ultra-sharp (H70h) and sharp (H60s) convolution kernels. The IMPAX EE “Extended Multiplanar Reconstruction Plugin” created simultaneous axial, sagittal, and coronary plane views. The respective distances were measured using the program’s built-in tool.

### Statistical methods

Power analysis was performed using G*Power 3.1.9.7 (Heinrich-Heine-Universität Düsseldorf, Germany)^25^. The sample size for the study group was chosen according to the number of individuals available at the time of the study. For the control group, the power analysis was exemplarily performed for a two-sample t-test (e.g., comparing treatment vs. control) with an effect size d = 0.4, a significance level α = 0.05, and a power = 0.8. The required sample size per group is approximately 82 individuals.

All collected data were analysed using Prism 10 Version 10.2.3 (GraphPad Software, San Diego, California, USA). The analysis included calculating and documenting the data’s mean, standard deviation, and confidence intervals. Descriptive evaluation and graphical representations were employed for a comprehensive overview. The Shapiro-Wilk test confirmed the data’s normal distribution, and then various statistical tests were applied.

Paired t-tests were used to check the distances between the left and right sides for normally distributed data to check between the left and right sides within the individuals. Wilcoxon matched-pairs signed rank test was used similarly to handle non-normally distributed data. To address the definition of symmetry, absolute values for the difference between the left and right sides were used, their mean and standard deviation calculated, and the symmetry was defined as a difference equal to or less than the mean plus one standard deviation.

The absolute differences between the left and right sides of the control and study groups were calculated. Again, the data was controlled for normal distribution by the Shapiro-Wilk test. Normally distributed data was compared by the unpaired t-test, and non-normally distributed data was compared by the Mann-Whitney test.

Contingency analysis was performed via Fisher’s exact test to examine the significance of the association of symmetry amongst the groups. The significance level was set at α = 0.05.

## Results

### Participants

Between November 2018 and December 2022, 195 individuals received an intraoperative CBCT scan after ORIF of a midfacial fracture. Bilateral fractures occurred in 13.3% of individuals (*n* = 26), and insufficient imaging quality was present in 19.0% of individuals (*n* = 37), leading to a study population of 132 individuals. After applying the inclusion and exclusion criteria, the control group comprised 82 individuals with an equal distribution of male and female individuals.

### Descriptive data

There were 85 male and 37 female individuals, resulting in a male-female ratio of 1.8 to 1 in the study group and 41 male and female individuals each in the control group.

The mean age of the study group was 49.8 ± 19.5 (95% CI 46.4 to 53.2) years. Male patients were 42.7 ± 16.1 (95% CI 39.3 to 46.2), and female patients were 62.6 ± 18.8 (95% CI 57.1 to 68.1) years old. Male and female patients in the study group had a significant age difference (Mann-Whitney test, *p* < 0.0001; difference between medians 20.0 years; U = 849.5).

The mean age of the control group was 52.1 ± 18.4 (95% CI 48.0 to 56.1) years. Male patients were 52.0 ± 15.6 (95% CI 47.0 to 56.9), and female patients were 52.2 ± 21.0 (95% CI 45.6 to 58.8) years old. There was no difference in age between male and female individuals in the control group (Mann-Whitney test, *p* = 0.9247; difference between medians 3.0 years; U = 830.0).

The overall age difference between the control and study groups was insignificant (Mann-Whitney test, *p* = 0.3104; difference between medians 6.5 years; U = 4964.5).

### Outcome data

**Fig. 4:**
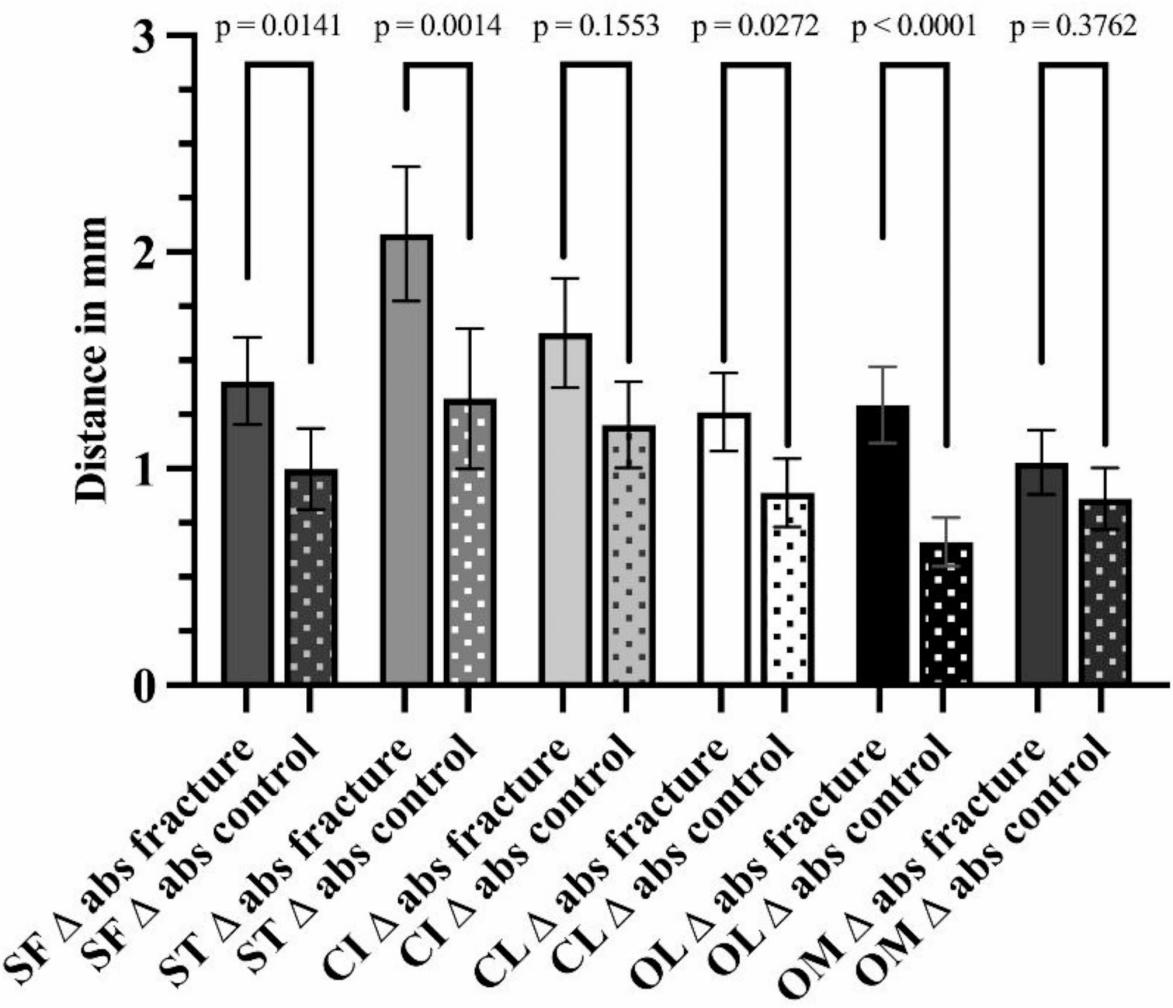
Absolute differences in the skeletal landmarks’ distances between the left and right sides of the study and the control groups. Depicted are the means, the whiskers show the 95% confidence interval of mean. The brackets indicate the p-values of the Mann-Whitney tests (SF = frontozygomatic suture, ST = temporozygomatic suture, CI = infraorbital canal, CL = crista lacrimalis posterior, OL = lateral orbita, OM = medial orbita, ∆ = difference, abs = absolute, fracture = study group, control = control group).

There was a significant difference for all measured skeletal landmarks between the left and right sides in the control and the study groups (Tables 1 and 2). In order to determine the degree of symmetry, the means of the absolute distances of difference were computed and used to evaluate the symmetry of the skeletal landmarks. There were significant differences in absolute distances between the study and control groups for all skeletal landmarks but the infraorbital canal and the medial orbita (Table 3; Fig. 4). The average of the absolute differences of all skeletal landmarks between the left and right sides were 1.5 ± 1.3 mm in the study group and 1.0 ± 0.9 mm in the control group and their medians presented statistically significant differences (Mann-Whitney test, *p* < 0.0001; difference between medians 0.3 mm; U = 156,343).

**Table 1:** Means for the skeletal landmarks and their significance in differences of the control group (*n* = 82).

Skeletal landmark	Mean ± SD Right side (mm)	95% CI Right side (mm)	Mean ± SD Left side (mm)	95% CI Left side (mm)	Paired t-test
Frontozygomatic suture	51.86 ± 2.40	51.33 to 52.39	51.25 ± 2.50	50.70 to 51.80	*p* < 0.0001, median of differences 0.45, W = 1531^†^
Temporozygomatic suture	59.55 ± 3.71	58.74 to 60.37	59.12 ± 3.38	58.37 to 59.86	*p* = 0.0436, mean of differences 0.44 ± 1.93 95% CI 0.01 to 0.86
Infraorbital canal	32.71 ± 2.32	32.20 to 33.22	32.94 ± 2.22	32.45 to 33.43	*p* = 0.1584, mean of differences − 0.23 ± 1.49 95% CI − 0.56 to 0.09
Crista lacrimalis posterior	16.85 ± 1.52	16.51 to 17.18	16.64 ± 1.76	16.25 to 17.02	*p* = 0.0964, mean of differences 0.21 ± 1.13 95% CI − 0.04 to 0.46
Lateral orbita	47.15 ± 2.27	46.66 to 47.65	46.86 ± 2.27	46.36 to 47.36	*p* = 0.0011, mean of differences 0.30 ± 0.79 95% CI 0.12 to 0.47
Medial orbita	13.82 ± 1.77	13.44 to 14.21	13.51 ± 1.77	13.10 to 13.90	*p* = 0.0084, mean of differences 0.31 ± 1.04 95% CI 0.08 to 0.54

^†^Wilcoxon matched-pairs signed rank test.

**Table 2:** Means for the skeletal landmarks and their significance in differences of the study group (*n* = 132).

Skeletal landmark	Mean ± SD right side (mm)	95% CI right side (mm)	Mean ± SD left side (mm)	95% CI left side (mm)	Paired t-test
Frontozygomatic suture	53.01 ± 2.65	52.55 to 53.47	52.24 ± 2.48	51.81 to 52.66	*p* < 0.0001, mean of differences 0.77 ± 1.66 95% CI 0.49 to 1.06
Temporozygomatic suture	61.01 ± 3.48	60.41 to 61.60	60.09 ± 3.64	59.47 to 60.72	*p* < 0.0001, mean of differences 0.91 ± 2.60 95% CI 0.47 to 1.36
Infraorbital canal	31.11 ± 2.81	30.63 to 31.59	30.26 ± 2.81	29.78 to 30.75	*p* < 0.0001, median of differences 0.70, W = 3424^†^
Crista lacrimalis posterior	16.80 ± 1.59	16.53 to 17.07	16.19 ± 1.50	15.93 to 16.45	*p* < 0.0001, mean of differences 0.61 ± 1.52 95% CI 0.35 to 0.87
Lateral orbita	47.23 ± 2.31	46.83 to 47.63	46.49 ± 2.26	46.10 to 46.88	*p* < 0.0001, mean of differences 0.73 ± 1.48 95% CI 0.48 to 0.99
Medial orbita	13.33 ± 3.41	12.75 to 13.92	12.96 ± 3.44	12.37 to 13.55	*p* = 0.0016, median of differences 0.10, W = 2330^†^

^†^Wilcoxon matched-pairs signed rank test.

**Table 3:** Means for the absolute differences of the skeletal landmarks of the control and study groups.

Skeletal landmark	Control group (*n* = 82)		Study group (*n* = 132)		Mann-Whitney test
	Mean ± SD (mm)	95% CI (mm)	Mean ± SD (mm)	95% CI (mm)	
Frontozygomatic suture	1.00 ± 0.85	0.81 to 1.19	1.40 ± 1.17	1.20 to 1.61	*p* = 0.0141; difference between medians 0.45; U = 4335
Temporozygomatic suture	1.32 ± 1.47	1.00 to 1.65	2.03 ± 1.80	1.77 to 2.39	*p* = 0.0014; difference between medians 0.65; U = 4011
Infraorbital canal	1.20 ± 0.90	1.01 to 1.40	1.63 ± 1.47	1.37 to 1.88	*p* = 0.1553; difference between medians 0.20; U = 4786
Crista lacrimalis posterior	0.89 ± 0.72	0.73 to 1.05	1.26 ± 1.05	1.08 to 1.44	*p* = 0.0272; difference between medians 0.20; U = 4442
Lateral orbita	0.66 ± 0.52	0.55 to 0.77	1.30 ± 1.02	1.12 to 1.47	*p* < 0.0001; difference between medians 0.50; U = 3355
Medial orbita	0.86 ± 0.65	0.72 to 1.00	1.03 ± 0.86	0.88 to 1.18	*p* = 0.3762; difference between medians 0.00; U = 5022

### Main results

The absolute differences in the skeletal landmarks were compared. When none or a maximum of one of the skeletal landmarks was not within range of the mean plus one standard deviation, the respective individual was considered to be symmetrical. In the control group, 66 individuals (80.5%) showed symmetry, whilst 16 individuals (18.5%) did not. The study group revealed 104 patients (78.8%) to be symmetrical and 28 patients (21.2%) not to be symmetrical. Even though there is a tendency towards an increased asymmetry in the study group, no relationship between the symmetry and the presence or absence of a midfacial fracture could be verified (Fisher’s exact test, *p* = 0.8624, odds ratio 1.11, 95% CI 0.55 to 2.16).

There was a strong association between fracture reduction in excess, or “overcorrection”, and the right side and between an insufficient reduction, or “undercorrection”, and the left side for all skeletal landmarks but the medial orbita (Table 4). Except for the infraorbital canal, all skeletal landmarks were further away from the median sagittal plane on the right side than on the left side (58.5 to 65.9% of individuals) in the control group.

**Table 4:** Number of individuals and their dependence between the fracture reduction and the side of fracture.

Skeletal landmark	Right side	Left side	Fisher’s exact test
Frontozygomatic suture			
Insufficient reduction	20	45	*p* = 0.0002 Odds ratio 0.23, 95% CI 0.11 to 0.49
Excess in reduction	35	18	
Temporozygomatic suture			
Insufficient reduction	28	47	*p* = 0.0053 Odds ratio 0.33, 95% CI 0.16 to 0.71
Excess in reduction	31	17	
Infraorbital canal			
Insufficient reduction	22	45	*p* = 0.0011 Odds ratio 0.29, 95% CI 0.13 to 0.61
Excess in reduction	36	21	
Crista lacrimalis posterior			
Insufficient reduction	21	47	*p* = 0.0002 Odds ratio 0.22, 95% CI 0.11 to 0.48
Excess in reduction	34	17	
Lateral orbita			
Insufficient reduction	16	44	*p* < 0.0001 Odds ratio 0.15, 95% CI 0.07 to 0.35
Excess in reduction	45	19	
Medial orbita			
Insufficient reduction	24	35	*p* = 0.2681 Odds ratio 0.62, 95% CI 0.31 to 1.31
Excess in reduction	31	28	

## Discussion

### Key results

The skeletal landmarks on the right side tend to be further away from the median sagittal plane in both the control and the study groups. Regarding the distances of the fractured compared to the healthy sides, there was a statistically significant dependence on excessive reduction on the right side and insufficient reduction on the left side, albeit no relationship between the symmetry and the presence or absence of a midfacial fracture could be verified.

ORIF of midfacial fractures with intraoperative three-dimensional imaging control employing a CBCT scan showed the same degree of symmetry as a control population. An overall median difference of 0.3 mm for all skeletal landmarks between the study and the control groups yielded clinical insignificance.

### Limitations

The study’s main drawback is that it utilizes two-dimensional parameters for analysis. There could be methodological drawbacks, such as the subjective determination of the median sagittal plane and the selection of the measurement points that may not capture the full complexity of facial asymmetry. However, the parameters were obtained from three-dimensional radiographs and have proven reliable before^23^.

Secondly, interpretative and contextual limitations could arise. Despite being based on three-dimensional imaging, linear measurements reduce spatial complexity to two-dimensional data, which can oversimplify the intricate three-dimensional relationships of the facial skeleton.

Third, biological variations with minor facial asymmetries are expected and may not have clinical significance. Usually, there is a fluent transition from a normal variation to a pathological asymmetry, and an absolute value is highly subjective.

Another drawback is a possible selection bias due to the retrospective study design. The issue was solved by including all patients with midfacial fractures who had received ORIF at our clinic for the study group and all symmetrical patients without a midfacial ailment for the control group within the respective study periods. Even though power analysis was performed, population-specific variations cannot be excluded, and the results may not be generalized across all populations due to differences in genetic background, age, and sex. Asymmetry norms vary geographically and demographically, and the study’s sample was drawn from Central Europe. Future studies could employ a prospective multi-centre study design and assess more geographical locations and various ethnicities, addressing this drawback.

### Interpretation

The primary indication for operative management of ZMC fractures is the ZMC or zygomatic arch displacement with accompanying functional and aesthetic alterations^26^. The main goal of ORIF is to restore normal facial contours and, thus, their form and symmetry^27^. Assurance of adequate reduction is performed by radiography, and recent developments caused a shift from postoperative two-dimensional radiographs to intraoperative three-dimensional radiographs^14–21^. Appropriate measurements were required to evaluate the reduction control.

Some publications dealt with the reduction control categorically^28^, while other studies quantified the reduction results^20,29^. The presented study aimed to quantify three-dimensional radiographs through clearly defined skeletal landmarks and compare these between the healthy and affected sides. JOHNER measured linear distances and angles of the reduced fracture and compared it to a mirrored model of the healthy side. However, there was no clearly defined anatomic location for the measurement of reduction control; instead, the maximum discrepancy was measured from the computed ideal position. Additionally, the study only dealt with zygomatic arch fractures and excluded ZMC fractures^20^, making it hard to compare the studies due to the different methodologies.

DUBRON segmented the zygomatic bones and orbitae, mirrored one of the sides and performed a volumetric matching according to the orbita. Three-dimensional measurements were performed, and a three-dimensional value was given via the mean absolute distance^29^. Whilst this kind of measurement heeds all three-dimensional aspects of fracture reduction, it does not verify in which precise location there may be a difference or in which direction, i.e., an “overcorrection” or “undercorrection” was found. Additionally, matching errors could root from an affection of the floor of the orbit, which is present in virtually all ZMC fractures except isolated zygomatic arch fractures.

A previous study analysed CT scans of healthy individuals for symmetry, employing a robust, reproducible, and readily performable methodology without the need for sophisticated hardware and software^23^. The simplicity of analysis led to this follow-up study to test for symmetry restoration in patients with ZMC fractures. Even though fewer individuals in the study group fell in the definition of symmetry in the presented study, there was no statistically significant dependence on the number of individuals and symmetry compared to a control group, concluding the achievement of satisfactory reduction after ORIF of ZMC fractures. This result contrasts the fact that there were statistical differences in the skeletal landmarks between the left and right sides of the study group and the differences in the absolute values between the study and control groups. A lower symmetry rate is found in other study groups, too^29^. There are also reports of inadequate reduction of much greater magnitude; however, the asymmetry threshold may be relatively low^30^.

Although a three-dimensional analysis to asses symmetry is deemed superior^22^, the assessment is intricate and requires advanced technology. Thus, immediate reduction verification could be performed on-site using simple methods and linear measurements^31,32^.

Other possible approaches for treating ZMC fractures assure symmetric reduction. For example, augmented reality could be employed during surgery, yielding excellent results of preoperatively planned positioning for ORIF^33^. Alternatively, patient-specific surgical guides could be designed and printed preoperatively, facilitating precise reduction of comminute ZMC fractures^34^.

A fascinating finding of the study was that the right sides tended to be “overcorrected”, whereas the fractures on the left sides tended to be “undercorrected”, which was connected to a statistically significant association. Interestingly, the control group showed larger values from the midline on the right side in all but one of the skeletal landmarks. The authors believe there are various possible explanations. One explanation could be an actual misaligned reduction and a true excess on the right side since the surgeon is usually positioned on the patient’s right side and could exert more force for fracture reduction. The scope of the study did not focus on the variability of surgeons performing the procedure, but a possible solution for this effect could be that the surgeon will perform the reduction on the side of the fracture, thereby assuring adequate force and control for reduction. Additionally, careful measurements of intraoperative CBCT scans could show the surgeon whether or not a correction needs to be performed before wound closure. Another explanation could be that larger values were factual on the right side, assuming an ideal reduction. The latter theory finds agreement within the literature that found a similar tendency in probands without apparent midfacial pathology^23,35^. The presence of geographical and chronological differences between populations could play another role in these peculiar findings^36^. Yet another possible cause could be the oversimplification of the chosen examination method. Future research employing three-dimensional methods for reduction control could illuminate this finding.

Even though three-dimensional methodology is recommended to assess symmetry^22^, the presented study did not use three-dimensional models but linear measurements in three-dimensional imaging. The reason for using linear measurements is simple: some studies may compare the left and right sides but instead, compare congruence between the segmented bodies instead of proper symmetry along a mirrored plane^31,37,38^. The authors believe this method is unsuitable for reduction control, which is consented to in the literature^39^. This problem can be overcome by modifying the study’s methodology^37383940^, i.e., by evaluating the symmetry of the zygomatic bone and its sutures using unaffected neighbouring bones^29,40^. Two ways of assessing symmetry were employed: linear measurements of the left and right sides from the median plane and congruence measurements between the segmented zygomas of the left and right sides. On average, there were differences between the left and right sides that ranged from 1.41 to 3.13 mm for all landmarks of the zygomatic bone, deeming the frontozygomatic suture as the landmark with the most minor variability and most highly conserved symmetry^41^. This study seems suitable for comparing the presented study’s values, and all the ascertained average distances are lower than those assessed by BELCASTRO. Nonetheless, the absolute differences in the skeletal landmarks between the left and right sides were significantly higher in the study group than in the control group in all but two landmarks. A similar outcome was elucidated in comparable studies^39,42^. Finally, even though the absolute distances of all skeletal landmarks between the left and right sides of the study and the control groups display statistical significance, the difference between medians coming to 0.3 mm is minute, contradicting a noteworthy clinical role.

After ORIF of ZMC fractures, the symmetry evaluation using intraoperative CBCT scans directly impacts functional and aesthetic recovery and is a key predictor of favourable surgical results. Intraoperative three-dimensional imaging is a valuable technique for fast fracture reduction and implant location assessment that improves surgical accuracy, minimises the need for further procedures, and enables immediate modifications. As a result, the patient is safer and further expenses may be prevented.

These results emphasise the value of using different techniques to evaluate symmetry in environments with limited resources where CBCT technology may not be readily available. Surgeons may use manual palpation methods, comprehensive preoperative planning, and any intraoperative two-dimensional imaging available. Training programs strongly emphasise developing these abilities and utilising inexpensive imaging techniques for the best results before relying on sophisticated technology. Another possibility is the use of remote surgical assistance systems, which might help close gaps in technology availability by sharing images for expert consultation.

By concentrating on these applicable ramifications, surgical procedures can be modified to prioritise symmetry, even in settings with little funding, thus enhancing patient outcomes globally.

### Generalisability

The population sizes for the control and study groups in the presented study are within the range of those in the literature^29,37,39,41^ or even exceed them^33,40^, deeming the presented data trustworthy and robust. Considering the limitations and possible drawbacks, the presented measurements are reproducible, easy to perform, and readily available, even to caregivers with less advanced technology, rendering the methodology an ideal tool for immediately assessing symmetry and reduction control.

Given the availability of three-dimensional imaging and relatively simple data processing, the methodology can be used for various surgical techniques and patient demographics and could also be applied to scientific studies to evaluate symmetry in the midfacial skeleton. Different teams can communicate better, allowing for a comparison of clinical and research results. It is a helpful tool to close the gap between sophisticated technology, less advanced imaging techniques and data processing availability, offering point-of-care to a broad spectrum of patients globally.

## Data Availability

The datasets generated during and analysed during the current study are available from the corresponding author upon reasonable request.
